# RNA-seq Reveals Dysregulation of Novel Melanocyte Genes upon Oxidative Stress: Implications in Vitiligo Pathogenesis

**DOI:** 10.1155/2019/2841814

**Published:** 2019-12-04

**Authors:** Konduru Seetharama Sastry, Haroon Naeem, Younes Mokrab, Aouatef Ismail Chouchane

**Affiliations:** ^1^Dermatology Research Unit, Division of Translational Medicine, Research Department, OPC 5th Floor, Sidra Medicine, Doha, PO Box 26999, Qatar; ^2^Human Genetics, Translational Medicine, OPC 5th Floor, Sidra Medicine, Doha, PO Box 26999, Qatar

## Abstract

Oxidative stress is known to induce melanocyte death, but the underlying mechanisms are incompletely understood. To identify oxidative stress-induced global gene expression changes in melanocytes, we treated PIG1 melanocytes with H_2_O_2_ in a dose- and time-dependent manner and performed RNA-seq. This approach allowed us to capture the events occurring early as well as late phase after treatment with H_2_O_2_. Our bioinformatics analysis identified differentially expressed genes involved in various biological processes of melanocytes which are known to contribute to the vitiligo development, such as apoptosis, autophagy, cell cycle regulation, cell adhesion, immune and inflammatory responses, melanocyte pluripotency, and developmental signaling such as WNT and NOTCH pathways. We uncovered several novel genes that are not previously described to be involved in melanocytic response to stress nor in vitiligo pathogenesis. Quantitative PCR and western blot analysis of selected proteins, performed on PIG1 and primary human epidermal melanocytes, confirmed the RNA-seq data. Interestingly, we discovered an aberrant regulation of several transcription factors that are involved in diabetes, neurological, and psychiatric diseases, all of which are comorbid conditions in patients with vitiligo. Our results may lead to a better understanding of the molecular mechanisms underlying vitiligo pathogenesis and help developing new drug targets for effective treatment.

## 1. Introduction

Reactive oxygen species (ROS) such as hydrogen peroxide (H_2_O_2_), superoxide anion radical, and hydroxyl radical are generated in the cells endogenously as well as through exposure to extrinsic factors. Under physiological conditions, cells can maintain the intracellular redox homeostasis by scavenging the ROS. However, excessive ROS production disrupts the redox homeostasis and damages the organelles and biomolecules, resulting in the manifestation of a variety of diseases including skin conditions. Thus, ROS can affect diverse biological processes through multiple mechanisms [1].

Skin interfaces with the environment and thus is a major source of ROS. Additionally, ROS are continuously generated during the melanogenesis process in epidermal melanocytes, and this excessive ROS can lead to melanocyte cell death, resulting in skin conditions such as vitiligo [2]. Vitiligo is a progressive skin condition in which functional melanocytes in the epidermis are stressed and selectively destroyed leading to the absence of melanin and a consequent skin depigmentation. Numerous hypotheses about the etiology of vitiligo have been proposed, but it remains unclear what causes damage or death of melanocytes.

There is a compelling evidence that increased production of ROS and their accumulation is one of the major reasons for the death of melanocytes in vitiligo [2]. Very high levels of H_2_O_2_ have been reported in the epidermis and serum of vitiligo patients [3, 4]. Compared to melanocytes from healthy individuals, those from vitiligo patients showed increased sensitivity and cell death to oxidative stress caused by UVB and chemicals [5]. These observations suggest that melanocytes in vitiligo skin are inherently sensitive to oxidative stress. Thus, identifying and targeting signaling pathways activated by ROS that are responsible for melanocyte death will help prevent the cell damage and probably restore melanocytes in the vitiliginous skin.

Although some of the pathways that underlie H_2_O_2_-induced transcriptional changes in melanocytes are known, a comprehensive view of stress-induced gene regulation is still elusive. While much of the understanding of ROS-induced signaling in melanocytes is based on single gene/pathway approaches [6, 7], studies on whole-transcriptomic approaches in melanocytes were performed using a single concentration/time point of oxidative stress-inducing agent [8]. Thus, the goal of the present study was to unravel, using RNA-seq, diverse differentially expressed genes (DEGs) in melanocytes that are modulated by H_2_O_2_-induced oxidative stress in a time- and dose-dependent manner.

## 2. Results and Discussion

### 2.1. Generation of Oxidative Stress in Melanocytes Using H_2_O_2_ and Its Detection

**Figure 1:**
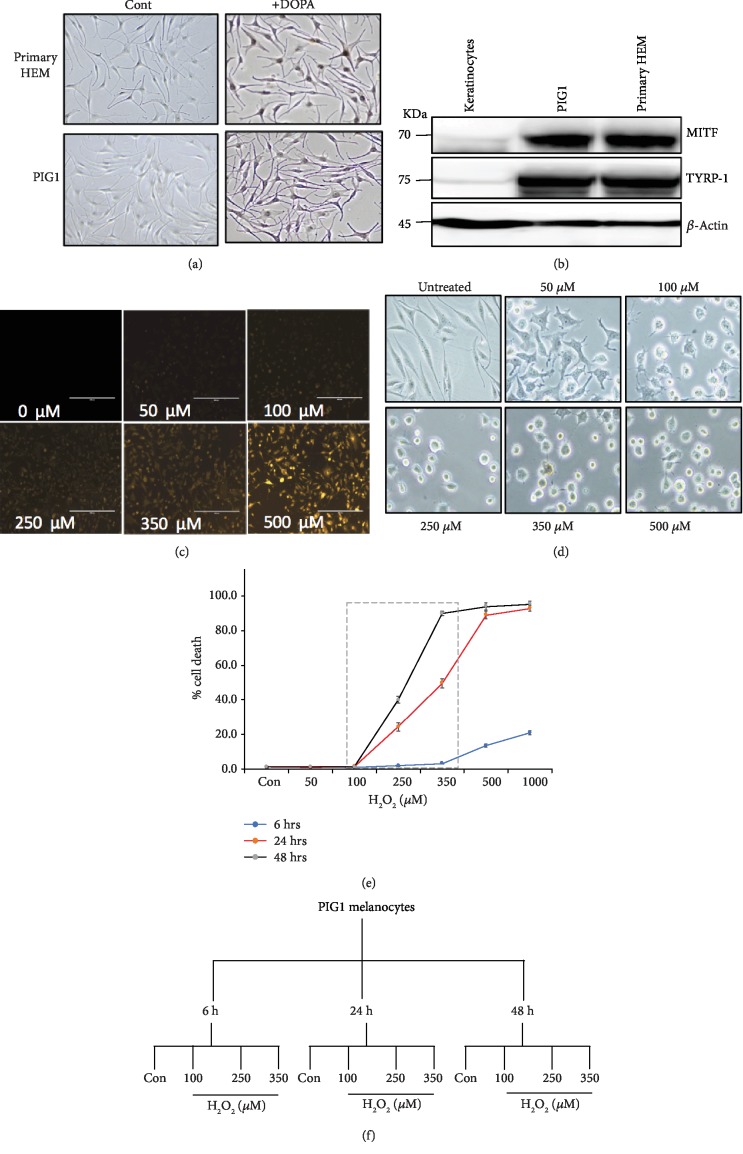
Characterization of melanocytes and H_2_O_2_-induced oxidative death: (a) PIG1 and primary HEM were incubated with L-DOPA or PBS (control) for 5 h and images were obtained using phase contrast microscopy. Note that the melanocytes are branched and stained black by L-DOPA, confirming the presence of DOPA oxidase (tyrosinase) activity. (b) Indicated cells were cultured, and cell lysates were probed with MITF and TYRP-1 antibodies using western blotting. Equal loading was confirmed using *β*-actin antibodies. (c) PIG1 melanocytes were either left untreated or incubated with indicated concentration of H_2_O_2_ for 2 h at 37°C. The oxidative stress was detected by staining cells with CellROX Orange dye for 30 min, and images were obtained by the Evos fluorescent microscope. (d) To observe the morphological changes, phase contrast microscopic images were obtained 3 h after exposure to H_2_O_2_. (e) PIG1 melanocytes were cultured with indicated concentration of H_2_O_2_ for 6 h, 24 h, and 48 h. Cell viability was checked by trypan blue dye exclusion assay. Mean (three independent observations) values of viability percentages were plotted at different time intervals. Greater reduction in cell viability was found with increasing concentration of H_2_O_2_. Grey box includes the time points chosen for RNA-seq experiments. (f) Flow chart showing the different time points and concentrations of H_2_O_2_ used in RNA-seq experiments.

Various batches of isolated primary human epidermal melanocytes (HEM) respond differently to same treatments with various drugs including H_2_O_2_. These limitations can be avoided by using immortalized human epidermal melanocyte (PIG1) cell line which is a well characterized and most widely used for the fundamental understanding of melanocyte biology [7, 9, 10]. PIG1 cells and primary HEM exhibited dendriticity and extensive DOPA oxidase (tyrosinase) activity and expressed melanocyte-specific marker TYRP-1 and a master regulator of melanocyte development MITF transcription factor, all of which are characteristic features of melanocytes (Figures 1(a) and 1(b)). As compared to untreated controls, H_2_O_2_ triggered the ROS production, in a concentration-dependent manner as shown by progressive elevation of orange signal in treated cells (Figure 1(c)). Within 3 h after exposure to as low as 100 *μ*M H_2_O_2_, cells start losing their dendriticity, a first hallmark of cell death process (Figure 1(d)).

### 2.2. H_2_O_2_-Induced Cell Death in PIG1 Melanocytes

To choose the right concentration of H_2_O_2_ and dosing time points for RNA-seq experiments, we measured the cytotoxicity of H_2_O_2_ in melanocytes by exposing them to increasing concentrations of H_2_O_2_ for various exposure times. Cell viability was found to decrease in an inversely correlated manner to both dose and time of H_2_O_2_ exposure (Figure 1(e)). Across all concentrations of H_2_O_2_, cell death was minimal at time point 6 h. While a significant decrease in cell viability to 25% (24 h) and 40% (48 h) was observed in the presence of 250 *μ*M H_2_O_2_, it was reduced by 50% and 90% with 350 *μ*M H_2_O_2_ at these respective time points. Beyond these concentrations and time points, no cell viability was observed. Thus, to determine changes in gene expression using RNA-seq, wherein all stages of death process (early, mid, and late stages) could be represented, we have chosen 100 *μ*M (nonlethal dose, generally seen in healthy cells), 250 *μ*M (moderately lethal dose), and 350 *μ*M (highly lethal) of H_2_O_2_ and time points of 6 h, 24 h, and 48 h and performed with biological triplicates (Figure 1(f)).

### 2.3. Gene Expression Pattern

**Figure 2:**
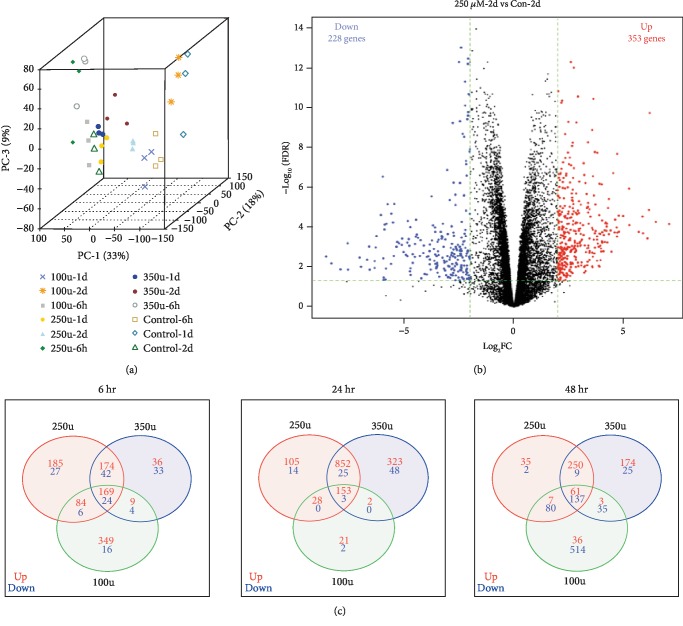
(a) MDS plot: multidimensional scaling plot shows the clustering of controls and H_2_O_2_ treated samples. A large disparity in expression of genes between controls and H_2_O_2_ treated with various doses and time points was clearly evident. (b) Volcano plot: the transcriptome of cells treated with 250 *μ*M H_2_O_2_ for 48 h was compared to control. The *x*-axis represents the log_2_ fold changes (log_2_FC), and the *y*-axis specifies the statistical significance (negative logarithm to the base 10 of FDR) of differential expression. Green vertical and horizontal lines reflect the filtering criteria (log_2_FC = ±2.0 and FDR = 0.05). Red and blue dots represent up- and downregulated genes, respectively. (c) The Venn diagram shows the number of genes upregulated (Red) or downregulated (blue) by treatment with H_2_O_2_ for 6 h (a), 24 h (b), and 48 h (c) compared to controls. The numbers of differentially regulated genes in two or three groups are shown in intersections.

MDS plot shows a clear clustering of controls and H_2_O_2_-treated samples (Figure 2(a)). The volcano plot (Figure 2(b)) shows an overall representation of RNA-seq results obtained with cells treated with 250 *μ*M H_2_O_2_ for 48 h. Similar pattern is observed with all other treatments (not shown).

### 2.4. H_2_O_2_ Alters Gene Expression in a Time- and Dose-Dependent Manner in Melanocytes

**Figure 3:**
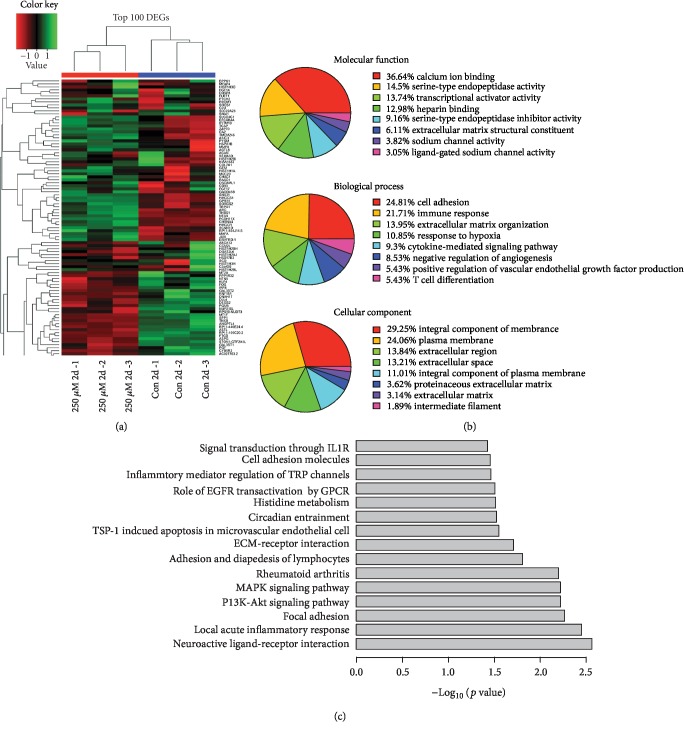
(a) The hierarchical clustering of top 100 differentially expressed genes between 250 *μ*M H_2_O_2_ at 48 h treatment and control. Red color is the upregulated and green color is the downregulated genes. (b) GO enrichment analysis of DEGs between 250 *μ*M H_2_O_2_ samples and control samples at 48 h according to the DAVID bioinformatics tool. (c) Biological pathway enrichment analysis of DEGs between 250 *μ*M H_2_O_2_ and control for 48 h using the DAVID bioinformatics tool.

H_2_O_2_ altered the expression changes in a time- and dose-dependent manner. We noticed a general trend that the number of genes induced by H_2_O_2_ exceeded the number of suppressed ones across time and concentrations (Figure 2(c)) (hypergeometric *p* value < 0.05 for DEGs between time points and concentrations). We noticed that there are significant overlapping DEGs between edgeR, DESeq2, and limma methods (Sup Fig-[Supplementary-material supplementary-material-1]).  Figure 3(a) shows the hierarchical clustering of top 100 DEGs between controls and 250 *μ*M H_2_O_2_ treatment, and Sup. Fig-[Supplementary-material supplementary-material-1] shows hierarchical clustering at other concentrations. The list of top 25 upregulated and downregulated genes (250 *μ*M, 48 h) is shown in Tables 1 and 2, respectively. While top upregulated genes are involved in calcium signaling, GPCR signaling, and growth and differentiation, top downregulated genes are involved in cytoskeleton, adhesion, immune mediators, and oxidation-reduction pathways.

### 2.5. GO Enrichment and Pathway Analysis

To better understand the functions of DEGs, we performed GO enrichment analysis using DAVID. At the level of molecular functions, the top 3 enrichment items included the ion binding, endopeptidase activity, and transcriptional activator activity (Figure 3(b) and Sup. Fig-[Supplementary-material supplementary-material-1]), whereas cell adhesion, immune response, and extracellular matrix organization were the most enriched in biological process. At the level of cellular component, the integral component of membranes and plasma membrane was top enriched. Pathways related to neuronal signaling, immune functions and inflammatory response, apoptosis/cell survival such as PI3K and MAPK pathways, cell adhesion, and migration are among the most relevant enriched ones (Figure 3(c), Sup. Fig-[Supplementary-material supplementary-material-1]). Similar results have been reported in previous studies that compared the differences in expression pattern in healthy vs. vitiligo skin [11].

Analysis of DEGs shows that the expression of certain genes known to be involved in signaling mechanisms contributing to various melanocytic processes is quite significant across time points in H_2_O_2_-treated cells. These were classified by their potential roles in cell death, cell cycle, melanogenesis, WNT signaling, etc. (Table 3). Since some of these genes are known to involve in more than one biological process, these were classified into several groups. Furthermore, the most interesting part of our study, we found several genes that are not previously described to be involved in melanocyte death or in vitiligo pathogenesis (Table 3). A detailed account of these novel genes and their possible role in melanocyte survival has been discussed in following sections.

### 2.6. Validation of RNA-seq Data

**Figure 4:**
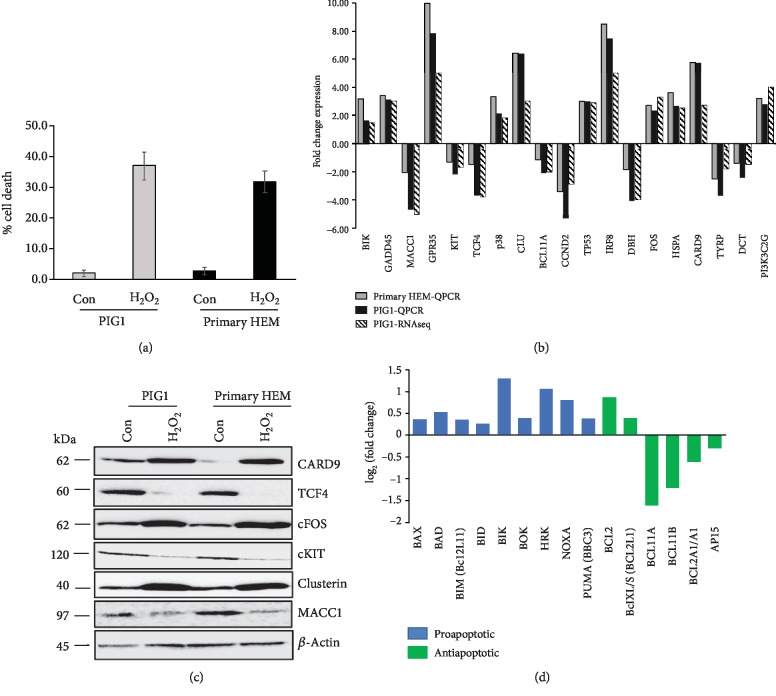
(a) PIG1 cells and primary HEM were treated with 250 *μ*M H_2_O_2_ for 48 h, and cell viability was measured by trypan blue dye exclusion assay. (b) PIG1 and primary HEM cells were treated as in (a), and mRNA extracted. Relative gene expression of indicated genes was measured using quantitative RT-PCR. GAPDH was used as internal control. (c) PIG1 cells and primary HEM were treated as in (a); cell lysates were collected and probed with indicated antibodies using western blotting. (d) The relative expression levels induced by treatment with 250 *μ*M H_2_O_2_ for 48 h of BH family proapoptotic (blue) and antiapoptotic (green) are shown. Only those members whose *p* value is <0.05 are shown.

We performed quantitative RT-PCR of selected genes on PIG1 melanocytes as well as primary HEM treated with 250 *μ*M H_2_O_2_ for 48 h, using GAPDH as a reference gene. At these conditions, the cell death in PIG1 and primary HEM cells was 36% and 31%, respectively (Figure 4(a)). We found an excellent agreement between the RNA-seq data and RT-PCR (Figure 4(b)). Similar results were observed when *β*-actin was used as a reference gene (Sup. Fig-[Supplementary-material supplementary-material-1]). Furthermore, we checked the expression of selected genes, *CRAD9*, *TCF4*, *FOS*, *KIT*, *CLU*, and *MACC1*, at protein level using the cell lysates of control and H_2_O_2_-treated PIG1 cells and primary HEM by western blotting. The expression patterns of these proteins (Figure 4(c)) confirm that there exists a good concordance between RNA-seq, RT-PCR, and western analysis, thus enhancing our confidence that melanocyte death is mediated by the differential molecular abnormalities of these proteins.

### 2.7. Cell Death Mechanisms

#### 2.7.1. Apoptosis

Apoptosis has been reported to be the main mechanism of melanocyte destruction by ROS in vitiligo [12–16]. Furthermore, previous GWAS studies identified that several candidate loci associated with vitiligo pathogenesis are regulators of apoptosis [17]. Apoptosis can occur through two distinct molecular pathways: intrinsic or extrinsic pathways.

**Figure 5:**
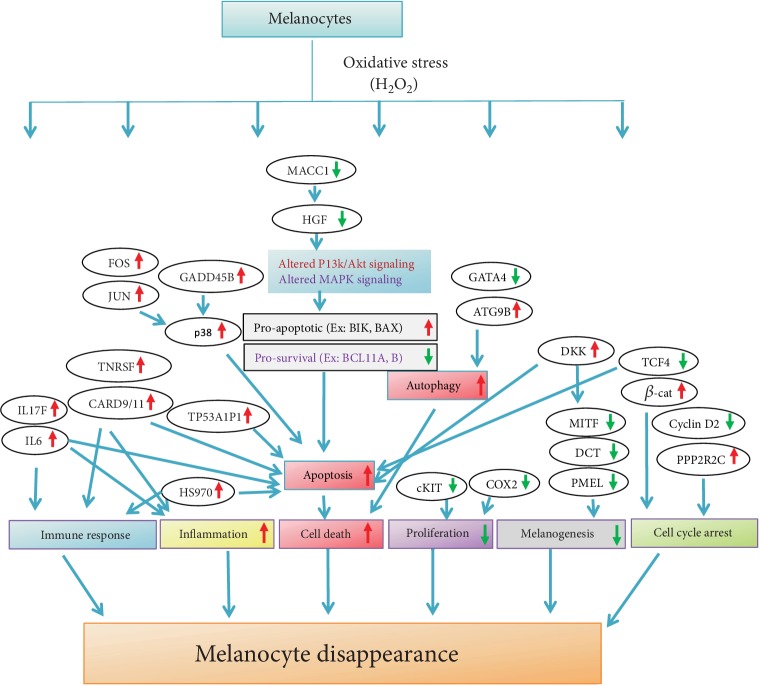
Flowchart depicts diverse signaling mechanisms altered by ROS in melanocytes. Red and green arrows indicate upregulation and downregulation of genes, respectively.

*(1) Intrinsic Pathway*. Lower expression of antiapoptotic proteins and elevated expression of proapoptotic proteins such as BAX and p53 have been noticed in vitiliginous skin as compared to normal skin [18]. For the first time, our RNA-seq results suggest that the regulation of apoptosis in stress-induced melanocytes is more complex than previously reported. Thus, while several proapoptotic proteins such as BAX, BAD, BIM, BID, BIK, BOK, HRK, NOXA, and PUMA were found to be upregulated in response to H_2_O_2_-induced stress, we found that the expression of antiapoptotic members such as BCL11A, BCL11B, A1, and API5 was suppressed (Figures 4(d) and 5). While differential expression of each of these members has a modest effect, simultaneous elevation of multiple proapoptotic genes and downregulation of several antiapoptotic genes will tilt the balance towards apoptosis in response to stress.

*(2) Extrinsic Apoptotic Pathway*. As far as extrinsic pathway is concerned, the members of the tumor necrosis factor receptor superfamily (TNFRSF) bind to death ligands TNFs. They are primarily involved in diverse biological processes such as immune homeostasis, execution of immune responses, inflammation, stimulation of apoptosis, and proliferation [19]. The most interesting observation from our study is that several members of TNFRSF such as TNFRSF-1B, 4, 8, 9, 10A, 11B, 12A, 13C, and 25 are significantly upregulated to various extent after treatment with H_2_O_2_ (Table 3, Sup. Table-[Supplementary-material supplementary-material-1]). The TNF-*α*, the ligand that binds to TNFRSF, has been indeed shown to accumulate in the skin and serum of vitiligo patients [20]. The overexpression of TNFRSF members may have a huge effect on cells. While one way, they can help execute immune responses against oxidative stress, on the other way, they activate melanocyte cell death.

#### 2.7.2. Autophagy

In addition to apoptosis, H_2_O_2_ also induced the expression of genes involved in autophagy. Of these, downregulation of a zinc finger TF, GATA4, is worth a mention. While silencing of GATA4 can trigger autophagy and apoptosis, overexpression of GATA4 elevated the gene expression of the survival proteins and suppressed the expression of other autophagy-related genes [21]. Suppression of GATA4 by H_2_O_2_ as seen in our study, an observation consistent with a previous report showing the downregulation of GATA3 in vitiligo melanocytes [22], may likely be responsible, at least partially, for the autophagic effects of H_2_O_2_. ATG9B, whose up expression was more prominent at 24 h after treatment with H_2_O_2_, is also well known to participate in autophagy [23]. Other autophagy genes either unchanged or downregulated may suggest that autophagy in H_2_O_2_-stressed melanocytes preferentially depend on GATA4 and ATG9B.

#### 2.7.3. Melanogenesis

In addition to the melanocyte death, abnormal melanogenesis is thought to contribute to the vitiligo pathogenesis. Consistent with previous studies [24, 25], we noticed a downregulation of several genes involved in pigmentation process, such as *TYRP1*, *PMEL*, *MLANA*, *DCT*, and *PLP1*, whose underexpression was more prominent at 48 h after treatment with H_2_O_2_ (Table 3), suggesting that these genes are aberrantly regulated by oxidative stress and play a role in disease pathology.

#### 2.7.4. Other Novel Cell Death Signaling Pathways

Besides classical BCL2 family members, numerous other proteins are known to control cell survival or death either directly or indirectly. For the first time, our study identified several such proteins and therefore it is worth discussing the most significant of them and their possible implications in melanocyte biology.

It has been shown that metastasis associated in colon cancer protein 1 (MACC1) can promote cell growth when overexpressed and promoted apoptosis in both in vitro and in vivo when underexpressed [26]. MACC1 activates the HGF/c-MET pathway, culminating in aberrant activation of multiple cellular responses such as proliferation, cell morphogenesis, migration, and breakdown of the extracellular matrix by altering Ras/MAPK and PI3K/Akt signaling pathways [27]. A key finding of the present study was that H_2_O_2_-induced oxidative stress significantly reduced the expression of both MACC1 and HGF in melanocytes (Table 3). This probably leads to the repression of Ras/MAPK and PI3K/Akt survival pathways, resulting in suppression of cell proliferation and induction of apoptosis (Figure 5). Further experiments are in progress to identify the underlying mechanism of MACC1/HGF-mediated apoptosis in melanocytes.

Growth Arrest and DNA Damage Inducible (GADD) family proteins are implicated in cell cycle arrest, apoptosis, innate immunity, and maintenance of genomic stability [28]. The transcription of GADD family proteins is induced by apoptotic cytokines and genotoxic stress. These proteins mediate apoptosis by activating the p38/JNK pathway. We discovered a concomitant upregulation of both GADD45B and p38 MAPK in melanocytes treated with H_2_O_2_ (Table 3), suggesting that at least part of the effects of GADD45 proteins on cell growth and apoptosis are mediated by activation of the p38 pathway.

Caspase Activation and Recruitment Domain (CARD) are protein interaction motifs found in a variety of proteins such as CARD9, CARD11, and CARD14. They are known to participate in activation or suppression of CARD containing members of the caspase family and play a pivotal regulatory role in cell apoptosis and inflammation [29]. We found that all these proteins are upregulated more significantly at 24 h after treatment with 250 *μ*M H_2_O_2_ (Sup. Table-[Supplementary-material supplementary-material-1]). This is consistent with a previous observation showing that CARD11 is upregulated in the Smyth line of chicken, which is an excellent avian model for human autoimmune vitiligo [30]. Similarly, variations in CARD7/NALP1 gene are associated with the development of generalized vitiligo, and its overexpression was demonstrated to induce apoptosis [31]. While NALP1 expression is only modestly increased, the expression of NALP3, another member of this family with similar function, is substantially increased in melanocytes exposed to H_2_O_2_ at 24 h (Sup. Table-[Supplementary-material supplementary-material-1]).

The expression of Tumor Protein p53-Regulated Apoptosis-Inducing Protein 1 (TP53AIP1) gene is inducible by p53 and is thought to play an important role in mediating p53-dependent apoptosis. An increased level of proapoptotic protein p53 was found in the lesional skin compared to perilesional or nonlesional areas in vitiligo patients [12]. In our study, a higher expression of TP53AIP1 was apparent at 48 h after treatment with H_2_O_2_, pointing to the fact that in addition to the direct involvement of aberrantly expressed BCL2 family proteins, the apoptosis in melanocytes could be induced by p53-dependent pathways.

#### 2.7.5. cKIT Expression, Heat Shock Response, and FOS/JUN Signaling

A modest decrease in the expression of c-KIT receptors in melanocytes treated with H_2_O_2_ was observed in our study. This may contribute to the melanocyte growth arrest and death, consistent with previous reports showing the downregulation of cKIT in the skin of vitiligo [24]. Furthermore, our results showed that stressed melanocytes expressed higher levels of HSPA1A/HSP70-1 and HSPA1B/HSP70-2 and FOS/JUN/p38 MAPK proteins, all of which can contribute to cell death (Figure 5).

The proapoptotic TNF signals are blocked by proteins that are induced by NF-*κ*B such as TNFR-associated factor 1 (TRAF1) [32]. Our results suggest that elevation of TRAF1 acts as an antiapoptotic mechanism to prevent death of melanocytes in response to stress. However, the enhanced expression of NFKB Inhibitor Zeta (NFKBIZ), an antagonist of NFKB, along with other proapoptotic proteins present in stressed cells, as observed in our study, possibly negate the protection offered by TRAF and ultimately ensues cell apoptosis.

#### 2.7.6. Cell Cycle Regulating Proteins

D-type cyclins (cyclin D1, D2, and D3) are well known to play critical roles in cell cycle progression by interacting with cyclin-dependent kinases, such as CDK4 and CDK6. High expression of cyclins has been detected in several tumors. Repression of cyclin D2 (CCND2) expression, and concomitant upregulation of PPP2R2C, that is implicated in the negative control of cell growth and division [33], as we observed in cells treated with 250 *μ*M H_2_O_2_ for 48 h, could result in the G1 arrest and subsequent growth retardation. The relatively unchanged or decreased expression of other cyclins may suggest that the arrest of the cell cycle progression in stressed cells preferentially depend on CCND2. Together, our results suggest that H_2_O_2_ induces cell cycle arrest by regulating the expressions of cell cycle-related proteins.

#### 2.7.7. Developmental/Pluripotency Pathways

Our RNA-seq analysis also revealed the modulation of proteins involved in developmental/pluripotency signaling, such as WNT, and NOTCH pathways. WNT5A, 5B, 6, 10B, and 11 and DKK1 are among the most differentially regulated ones by treatment with H_2_O_2_. WNT signaling is critical for the development of melanocyte and was shown to be affected in vitiligo skin [24]. Upregulation of WNT pathway inhibitor, DKK1 in our study was most impressive. This is because while incubation of melanocytes with DKK1 resulted in increased apoptosis and reduced pigmentation, overexpression of DKK1 was previously shown to suppress melanocyte function and growth by reducing the expression levels of MITF, DCT, tyrosinase, and PMEL [34]. This is consistent with our findings as we demonstrated that the expression levels of all these genes were indeed repressed. Thus, in addition to activating pathways that are directly involved in melanocyte death, H_2_O_2_ is also inhibiting the regeneration of melanocytes by altering the signaling mechanisms involved in melanocyte development.

Transcription factor 4 (TCF4) can control the nuclear response to Wnt/*β*-catenin signaling and also functioning of immune system cells, neurons, and melanocytes. Knockdown of TCF4 has been shown to induce cell cycle arrest and apoptosis [35]. Since significant suppression of TCF4 by H_2_O_2_ was evident from our study, it might be possible that TCF4-mediated cell cycle arrest and apoptosis form another layer of regulatory circuitry for the destruction of melanocyte under oxidative stress or in vitiligo condition. Other proteins that we found differentially regulated by H_2_O_2_ include ZEB1, SNAI1, MYT1L, SOX21, KLF4, and GRHL2, all of which are well known to control epithelial to mesenchymal transition, development, or pluripotency.

#### 2.7.8. Inflammation and Immune Signaling

Interleukin-17 is a family of six cytokines that includes IL-17A through IL-17F, which are well characterized for their roles in immune modulation. Increased expression of IL17 in the serum and skin of vitiligo patients has been reported [36]. In our present work, we found that among IL17 family cytokines, IL17F is substantially upregulated in melanocytes treated with H_2_O_2_. This upregulation can antagonize melanogenesis and also promote melanocyte death by downregulating BCL2 family proteins, an observation consistent with previous reports [37]. Similarly, serum concentration of IL6 is elevated in vitiligo patients [38], and our results confirmed IL6 overexpression at a cellular level. IL6 can induce the expression of cell adhesion molecules in melanocytes, thereby facilitating the interaction of melanocytes with immune cells and possibly induces B-cell activation, increasing the autoantibody production and subsequent damage of melanocytes [39]. In addition to IL6, our results show that several other proteins involved in immunity and inflammation, such as CXCL17 (-4.7), IL1A (-4.8), IL1B (-4.7), IL1RN (-3.5), and OASL (-1.8), have been found to be aberrantly expressed in stressed melanocytes [40].

#### 2.7.9. Transcription Factors

Transcription factors are critical for the transcriptional regulation of gene expression and play a key role in many biological processes. Therefore, we analyzed our RNA-seq data to obtain aberrantly regulated TFs in response to oxidative stress. We identified 30 upregulated and 18 downregulated TFs (Table 4). Some of these differentially regulated TFs play a role in immune system, pluripotency, differentiation, development, and cell death. A novel finding from our study is the aberrant expression in stressed melanocytes of TFs that play a role in diabetes, neurological, and psychiatric conditions, all of which are potential comorbid diseases in patients with vitiligo [41–43], but no role of these TFs has been previously reported in melanocytes.

Our findings shed light for the first time on this comorbid relationship: the TFs differentially expressed in stressed melanocytes might be the link between vitiligo and potentially associated conditions. This can be partially explained by the fact that although melanocytes are mainly found in the skin, they are also recognized in other parts of the body, including the eyes, ears, heart, and central nervous system where they are thought to have different roles from that played in the skin [42], but they may react in a similar way to stress. Another explanation is that the skin and brain share the same embryonic ectodermal origin, influenced by same hormones and neurotransmitters, and thus may have a similar behavior to stress. Additionally, nerve, pancreatic, and cardiac cells, like melanocytes, are vulnerable to ROS, and overproduction of ROS is shown to cause several diseases such as diabetes, rheumatoid arthritis, cardiovascular diseases, stroke, cancer, and other degenerative diseases in humans. It could be postulated that melanocytes, nerve, or pancreatic cells may respond to the oxidative stress by activation of common set of TFs, leading to the regulation of certain genes responsible for the occurrence of comorbid diseases. For example, myelin transcription factor 1-like (MYT1L) is known to be specifically expressed in the brain, and its dysregulation was shown to cause intellectual disability like autism spectrum disorders [44]. Similarly, ISL1 (ISL LIM homeobox 1) plays an important role in regulating insulin gene expression, as well as in the development of pancreatic cell lineages and motor neuron generation [45, 46]. Mutations in ISL1 have been associated with maturity onset diabetes of the young and type-2 diabetes [47]. Our results show that both these TFs are differentially regulated in stressed melanocytes, suggesting that they have a role not only in nerve/pancreatic cells as previously reported but also in melanocytes. A functional cross talk between the melanocyte, nervous, and immune systems can be another explanation for the occurrence of comorbid conditions, but further investigation is needed to clarify a potentially shared etiopathogenesis.

The most striking disadvantage of profiling the vitiligo skin being the lack of melanocytes in lesional skin, thus the actual pathways operated in melanocytes that are responsible for their disappearance remain uncovered. This study tries to fulfill the knowledge gaps encountered in previous studies and to identify novel genes or pathways that play a profound role in vitiligo pathogenesis which may have been missed in earlier studies.

Our study is not without limitations. It is always ideal to study gene expression using 3D skin models, which takes into account the effects of microenvironment. However, various technical challenges such as difficulty in quantifying melanocyte death in a mixed population of cells and difficulty in sorting the stressed melanocytes that are undergoing various cell death processes from a 3D skin model prohibit the use of a 3D model.

In summary, our RNA-seq approach identified potential novel melanocyte genes induced by oxidative stress in a dose-and time-dependent fashion. Novel findings from our study include the notable changes in the expression of diverse genes known to play a role in death and several functions in other cell types, but their role in melanocyte biology or death was not previously described. Thus, basing on the current study, it is reasonable to hypothesize that ROS-induced melanocyte damage is regulated by a complex network of diverse, fail-proof, multilayered signaling mechanisms (Figures 4 and 5). Thus, a multipronged approach is needed to effectively counter ROS effects and to prevent melanocyte loss in conditions like vitiligo. Identification of these novel genes provides an additional clue to vitiligo pathogenesis and opens new avenues for further investigation in melanocyte biology. Further studies are needed to unveil the precise function of these genes, which may help develop new drug targets in conditions associated with melanocyte stress such as in vitiligo.

## 3. Materials and Methods

### 3.1. Cell Lines and Reagents

PIG1 melanocyte cell line was a gift from Caroline Le Poole, Northwestern University, Chicago, Illinois, USA. Primary HEM, melanocyte culture medium M254, Human Melanocyte Growth Supplement (HMGS), keratinocyte medium M154, and Human Keratinocyte Growth Supplement (HKGS) were obtained from Thermo Fisher Scientific, USA. Keratinocyte cell line HaCaT was from AddexBio (USA). All antibodies and chemicals, unless specified, were obtained from Cell Signaling Technology (Beverly, MA, USA) and Sigma (Milwaukee, WI, USA), respectively.

### 3.2. Cell Culture and L-DOPA Staining

PIG1 and primary HEM were cultured in M254 medium supplemented with HMGS, at 37°C in a humidified incubator of 5% CO_2_. HaCaT cells were cultured in M154 medium supplemented with HKGS. L-DOPA staining of melanocytes was performed as described previously [48].

### 3.3. Cell Viability Assay and RNA Isolation

Cells were cultured in 10 cm BioCoat tissue culture plates (Corning) until 70% confluence. Then, medium was changed to supplement-free M254 medium and treated with various doses of H_2_O_2_ for various time points. At the end of incubation, both dead and live cells were collected, and cell viability was measured by trypan blue dye exclusion assay. Remaining cells were used to isolate DNA and RNA using the Total DNA/RNA isolation kit (Qiagen). All cytotoxicity data was representation of three independent experiments in triplicates. The quality and quantity of RNA were measured by OD A260/A280 by NanoDrop.

### 3.4. Measurement of ROS

ROS production was assessed as per manufacturer's instructions. In brief, cells were incubated with 10 *μ*M of CellROX Orange dye (Molecular Probes) for 30 min at 37°C, followed by washing twice with PBS. On contact with ROS, the fluorescein is oxidized and emits an orange fluorescence. Cells were viewed and imaged using EVOS fluorescence microscopy.

### 3.5. RT-PCR and Western Blotting

About 2 *μ*g of RNA was reverse transcribed to generate cDNA using random primers and Superscript III reverse transcriptase (Invitrogen). Quantitative RT-PCR reactions were carried out in triplicates on a QuantStudio 12K Flex Real Time PCR machine. The relative expression of each gene was calculated using the 2-DDCT method with GAPDH as reference. The primers were obtained from Integrated DNA Technologies, and their sequences can be obtained upon request. Fifty microgram of total protein was loaded on 10% SDS-PAGE gel, and standard western blotting protocol was used to detect proteins.

### 3.6. RNA-seq Data Analysis

Raw paired-end (PE) reads were adapter-trimmed using Trimmomatic [49]. To maximize the number of reads on mRNA, we filtered raw reads against the Human rRNA databases using the SortMeRNA tool [50]. Then, reads were filtered for vector sequences by mapping to NCBI UniVec database using SeqyClean [51]. Then, the reads were aligned to the GRCh37 reference genome using the STAR aligner [52] with default parameters for PE reads, and gene counts were generated using the Subread package tool featureCounts [53]. The limma/voom R Bioconductor packages were used for normalization of read counts and identification of DEGs between treatment and control groups [54]. GO enrichment and pathway analysis of DEGs were performed using the DAVID bioinformatics tool [55]. To identify DEGs, we set a threshold of absolute log_2_ fold change (log_2_FC) > 2.0 and FDR < 0.05. Furthermore, we used different platforms like edgeR and DEseq2 methods to confirm DEGs. We also statistically compared the DEGs between different contrast groups (time points for each concentration) using hypergeometric tests.

### 3.7. Statistical Analysis

Unless indicated otherwise, data represent the results for assays performed in triplicate, with error bars to S.D.

## Figures and Tables

**Table 1:** List of top 25 most upregulated genes by treatment with 250 *μ*M H_2_O_2_ for 48 h. These genes passed the FDR threshold < 0.05 and abs.log_2_FC of 2.

Gene_ID	Gene symbol	Gene name	Log_2_ fold change
ENSG00000120903	CHRNA2	Cholinergic receptor nicotinic alpha 2 subunit	7.1
ENSG00000100095	SEZ6L	Seizure related 6 homolog like	6.5
ENSG00000198576	ARC	Activity regulated cytoskeleton associated protein	6.2
ENSG00000124253	PCK1	Phosphoenolpyruvate carboxykinase 1	6.2
ENSG00000137270	GCM1	Glial cells missing homolog 1	6.1
ENSG00000184647	PRSS55	Serine protease 55	5.9
ENSG00000226364	AC102948.2	No description	5.9
ENSG00000182870	GALNT9	Polypeptide N-acetylgalactosaminyltransferase 9	5.7
ENSG00000087237	CETP	Cholesteryl ester transfer protein	5.3
ENSG00000142233	NTN5	Netrin 5	5.3
ENSG00000168081	PNOC	Prepronociceptin	5.0
ENSG00000172421	EFCAB3	EF-hand calcium binding domain 3	4.8
ENSG00000172137	CALB2	Calbindin 2	4.8
ENSG00000182896	TMEM95	Transmembrane protein 95	4.7
ENSG00000178623	GPR35	G protein-coupled receptor 35	4.7
ENSG00000185739	SRL	Sarcalumenin	4.7
ENSG00000178297	TMPRSS9	Transmembrane serine protease 9	4.7
ENSG00000136099	PCDH8	Protocadherin 8	4.6
ENSG00000225899	FRG2B	FSHD region gene 2 family member B	4.6
ENSG00000167772	ANGPTL4	Angiopoietin like 4	4.5
ENSG00000168746	C20orf62	Uncharacterized protein C20orf62	4.5
ENSG00000164266	SPINK1	Serine peptidase inhibitor, Kazal type 1	4.4
ENSG00000188886	ASTL	Astacin-like metalloendopeptidase	4.3
ENSG00000156466	GDF6	Growth differentiation factor 6	4.3
ENSG00000115353	TACR1	Tachykinin receptor 1	4.3

**Table 2:** List of top 25 most downregulated genes by treatment with 250 *μ*M H_2_O_2_ for 48 h. These genes passed the FDR threshold < 0.05 and abs.log_2_FC of 2.

Gene ID	Gene symbol	Gene name	Log_2_ fold change
ENSG00000205420	KRT6A	Keratin 6A	-8.6
ENSG00000171345	KRT19	Keratin 19	-8.3
ENSG00000163220	S100A9	S100 calcium-binding protein A9	-7.9
ENSG00000147256	ARHGAP36	Rho GTPase activating protein 36	-7.6
ENSG00000148346	LCN2	Lipocalin 2	-7.6
ENSG00000086548	CEACAM6	Carcinoembryonic antigen related cell Adhesion molecule 6	-7.0
ENSG00000168542	COL3A1	Collagen type III alpha 1 chain	-7.0
ENSG00000057149	SERPINB3	Serpin family B member 3	-6.7
ENSG00000087916	SLC6A14	Solute carrier family 6 member 14	-6.6
ENSG00000124107	SLPI	Secretory leukocyte peptidase inhibitor	-6.6
ENSG00000173432	SAA1	Serum amyloid A1	-6.4
ENSG00000186081	KRT5	Keratin 5	-6.4
ENSG00000124102	PI3	Protease inhibitor 3, skin-derived	-6.3
ENSG00000164107	HAND2	Heart and neural crest derivatives expressed 2	-6.3
ENSG00000181541	MAB21L2	Mab-21 like 2	-6.1
ENSG00000125810	CD93	CD93 molecule	-6.0
ENSG00000127954	STEAP4	STEAP4 metalloreductase	-6.0
ENSG00000158578	ALAS2	5′-Aminolevulinate synthase 2	-5.9
ENSG00000162896	PIGR	Polymeric immunoglobulin receptor	-5.9
ENSG00000016082	ISL1	ISL1 transcription factor, LIM/homeodomain	-5.9
ENSG00000186832	KRT16	Keratin 16	-5.9
ENSG00000125999	BPIFB1	BPI fold containing family B member 1	-5.9
ENSG00000129538	RNASE1	Ribonuclease 1	-5.8
ENSG00000171401	KRT13	Keratin 13	-5.8
ENSG00000166482	MFAP4	Microfibril-associated protein 4	-5.8

**Table 3:** Functional classes of selected differentially expressed genes with 250 *μ*M H_2_O_2_ for 48 h. These genes passed the FDR threshold < 0.05 and abs.log_2_FC of 2.

Putative function	Genes	Log_2_ fold change
Apoptosis	GADD45B	+2.6
	MACC1	-5.2
	HGF	-2.6
	GATA4	-2.0
	TP53AIP1	+1.9
	FOS	+2.6
	JUN	+2.1
	JUNB	+1.2
	FOSB	+2.6
	CARD9	+1.9
	ANXA3	-2.7
	IRF8	+3.7
	TCF4	-5.5
	HSPA1A	+2.0
	HSPA1B	+2.2
	CLU	+3.2
	TRAF1	+3.0
	TNFRSF4	+1.3
	TNFRSF12A	+1.5
	TNFRSF13C	+1.8
	GZMM	+3.5

Autophagy	GATA4	-2.0
	ATG9B	+1.7

Melanogenesis	TYRP1	-1.6
	PMEL	-1.4
	PLP1	-1.8
	HGF	-3.8

Cell cycle	PPP2R2C	+1.9
	CCND2	-3.0
	CDKN1A/p21	+0.7

WNT signaling	DKK1	+1.4
	TCF4	-5.5
	CTNNB1 (b-catenin)	-0.6

Adhesion	MACC1	-5.2
	CDH1/E cadherin	-1.8
	PTGS2	-1.4

Stress response	HSPA1A	+2.0
	HSPA1B	+2.2

Immunity	IL1B	-4.7
	IL6	+2.1
	IL17F	+3.3
	CXCL10	-5.0
	CCL2	-4.0
	CLU	+3.2
	TNFRSF4	+1.3
	TNFRSF12A	+1.5
	TNFRSF13C	+1.8
	TNFRSF25	+1.3
	IRF8	+3.7
	OASL	-1.8

- = downregulated; + = upregulated genes.

**Table 4:** Differentially regulated transcription factors between control and 250 *μ*M H_2_O_2_ for 48 h and their putative functions. These genes passed the FDR threshold < 0.05 and abs.log_2_FC of 2.

Putative function	Genes	Log_2_ fold change
Neuronal/brain development/psychiatric/mental retardation	POU3F1	+4.0
	MYT1L	+3.8
	DLX3	+3.7
	PITX3	+3.4
	FOXJ1	+3.3
	NHLH1	+2.9
	EGR3	+2.8
	ZNF804B	+2.2
	SOX21	+2.2
	GBX1	+2.0
	BARHL2	+2.0
	TCF4	-5.5
	ZFHX4	-3.4
	MEIS2	-3.1
	SETBP1	-2.6
	TFAP2B	-2.2

Immune system	IRF8	+3.7
	FOXN1	+3.5
	TBX21	+2.7
	IKZF1	-5.3
	IRF6	-2.1

Pluripotency/EMT	MYT1L	+3.8
	SNAI1	+3.6
	SOX21	+2.2
	KLF4	+2.0
	ZEB1	-5.5
	TCF4	-5.5
	GRHL2	-2.3

Growth/proliferation/differentiation/development	FOXN1	+3.5
	SP6	+3.5
	NHLH1	+2.9
	FOXL1	+2.8
	EGR3	+2.8
	FOSB	+2.6
	EGR1	+2.5
	SOX21	+2.2
	OVOL1	+2.1
	EHF	-5.6

Insulin/diabetes	MAFA	+3.7
	INSM2	+2.2
	BARHL2	+2.0
	ISL1	-5.9

Cardiovascular	GATA5	+3.0
	HAND2	-6.2
	MEIS2	-3.1

Metabolism	FOXL1	+2.8

Skin development	OVOL1	+2.1
	KLF4	+2.0

Cell death/survival	SNAI1	+3.6
	CSRNP3	-2.8
	IRF6	-2.1

- = downregulated; + = upregulated genes.

## Data Availability

The RNA-seq data used to support the findings of this study are available from the corresponding author upon request.

## Supplementary Materials

Supplementary Materials Supplementary Figure-1: comparison of the DEGs detected by three methods: Venn diagram of DEGs detected by the edgeR, DESeq, and limma. Supplementary Figure-2: the hierarchical clustering of top 100 differentially expressed genes (A): control vs. 100 *μ*M H_2_O_2_ (B): control vs. 350 *μ*M H_2_O_2_ at 48 h treatment and control. Red color is the upregulated and green color is the downregulated genes. Supplementary Figure-3: GO enrichment analysis of DEGs (A): 100 *μ*M H_2_O_2_ and control samples at 48 h; (B): 350 *μ*M H_2_O_2_ and control samples at 48 h, according to the DAVID bioinformatics tool. Supplementary Figure-4: biological pathway enrichment analysis of DEGs (A): 100 *μ*M H_2_O_2_ and control for 48 h; (B): 350 *μ*M H_2_O_2_ and control for 48 h using the DAVID bioinformatics tool. Supplementary Figure-5: PIG1 cells were treated with 250 *μ*M H_2_O_2_ for 48 h, and cell viability was measured by trypan blue dye exclusion assay (A). Relative gene expression of indicated genes was measured using quantitative RT-PCR (B). GAPDH and beta-actin were used as internal controls separately. Supplementary Table-1: list of differentially regulated selected apoptotic proteins after 24 h of treatment with H_2_O_2_.

## References

[1] Schieber M., Chandel N. S. (2014). ROS function in redox signaling and oxidative stress. *Current Biology*.

[2] Denat L., Kadekaro A. L., Marrot L., Leachman S. A., Abdel-Malek Z. A. (2014). Melanocytes as instigators and victims of oxidative stress. *The Journal of Investigative Dermatology*.

[3] Jain A., Mal J., Mehndiratta V., Chander R., Patra S. K. (2011). Study of oxidative stress in vitiligo. *Indian Journal of Clinical Biochemistry*.

[4] Schallreuter K. U., Moore J., Wood J. M. (1999). In vivo and in vitro evidence for hydrogen peroxide (H_2_O_2_) accumulation in the epidermis of patients with vitiligo and its successful removal by a UVB-activated pseudocatalase. *The Journal of Investigative Dermatology Symposium Proceedings*.

[5] Jimbow K., Chen H., Park J. S., Thomas P. D. (2001). Increased sensitivity of melanocytes to oxidative stress and abnormal expression of tyrosinase-related protein in vitiligo. *The British Journal of Dermatology*.

[6] Arowojolu O. A., Orlow S. J., Elbuluk N., Manga P. (2017). The nuclear factor (erythroid-derived 2)-like 2 (NRF2) antioxidant response promotes melanocyte viability and reduces toxicity of the vitiligo‐inducing phenol monobenzone. *Experimental Dermatology*.

[7] Mosenson J. A., Flood K., Klarquist J. (2014). Preferential secretion of inducible HSP70 by vitiligo melanocytes under stress. *Pigment Cell & Melanoma Research*.

[8] Yang G., Zhang G., Pittelkow M. R., Ramoni M., Tsao H. (2006). Expression profiling of UVB response in melanocytes identifies a set of p53-target genes. *The Journal of Investigative Dermatology*.

[9] Le Poole I. C., van den Berg F. M., van den Wijngaard R. M. (1997). Generation of a human melanocyte cell line by introduction of HPV16 E6 and E7 genes. *In Vitro Cellular & Developmental Biology-Animal*.

[10] Zhou Z., Li C. Y., Li K., Wang T., Zhang B., Gao T. W. (2009). Decreased methionine sulphoxide reductase A expression renders melanocytes more sensitive to oxidative stress: a possible cause for melanocyte loss in vitiligo. *The British Journal of Dermatology*.

[11] Wang P., Li Y., Nie H. (2016). The changes of gene expression profiling between segmental vitiligo, generalized vitiligo and healthy individual. *Journal of Dermatological Science*.

[12] Abdel-Aal A. M., Kasem M. A., Abdel-Rahman A. H. (2002). Evaluation of the role of apoptosis in vitiligo: immunohistochemical expression of P53, Bcl-2 and MART-1 antigens. *The Egyptian Journal of Hospital Medicine*.

[13] Huang C. L., Nordlund J. J., Boissy R. (2002). Vitiligo: a manifestation of apoptosis?. *American Journal of Clinical Dermatology*.

[14] Kumar R., Parsad D. (2012). Melanocytorrhagy and apoptosis in vitiligo: connecting jigsaw pieces. *Indian Journal of Dermatology, Venereology and Leprology*.

[15] Rodrigues M., Ezzedine K., Hamzavi I., Pandya A. G., Harris J. E., Vitiligo W. G. (2017). New discoveries in the pathogenesis and classification of vitiligo. *Journal of the American Academy of Dermatology*.

[16] Le Poole I. C., Wankowicz-Kalinska A., van den Wijngaard R. M., Nickoloff B. J., Das P. K. (2004). Autoimmune aspects of depigmentation in vitiligo. *The Journal of Investigative Dermatology Symposium Proceedings*.

[17] Jin Y., Andersen G., Yorgov D. (2016). Genome-wide association studies of autoimmune vitiligo identify 23 new risk loci and highlight key pathways and regulatory variants. *Nature Genetics*.

[18] Lee A. Y., Youm Y. H., Kim N. H., Yang H., Choi W. I. (2004). Keratinocytes in the depigmented epidermis of vitiligo are more vulnerable to trauma (suction) than keratinocytes in the normally pigmented epidermis, resulting in their apoptosis. *The British Journal of Dermatology*.

[19] Mak T. W., Yeh W. C. (2002). Signaling for survival and apoptosis in the immune system. *Arthritis Research*.

[20] Birol A., Kisa U., Kurtipek G. S. (2006). Increased tumor necrosis factor alpha (TNF-*α*) and interleukin 1 alpha (IL1-*α*) levels in the lesional skin of patients with nonsegmental vitiligo. *International Journal of Dermatology*.

[21] Suzuki Y. J. (2011). Cell signaling pathways for the regulation of GATA4 transcription factor: implications for cell growth and apoptosis. *Cellular Signalling*.

[22] Stromberg S., Björklund M. G., Asplund A. (2008). Transcriptional profiling of melanocytes from patients with vitiligo vulgaris. *Pigment Cell & Melanoma Research*.

[23] Wang N., Tan H. Y., Li S., Feng Y. (2017). Atg9b deficiency suppresses autophagy and potentiates endoplasmic reticulum stress-associated hepatocyte apoptosis in hepatocarcinogenesis. *Theranostics*.

[24] Regazzetti C., Joly F., Marty C. (2015). Transcriptional analysis of vitiligo skin reveals the alteration of WNT pathway: a promising target for repigmenting vitiligo patients. *The Journal of Investigative Dermatology*.

[25] Yu R., Broady R., Huang Y. (2012). Transcriptome analysis reveals markers of aberrantly activated innate immunity in vitiligo lesional and non-lesional skin. *PLoS One*.

[26] Yao Y., Dou C., Lu Z., Zheng X., Liu Q. (2015). MACC1 suppresses cell apoptosis in hepatocellular carcinoma by targeting the HGF/c-MET/AKT pathway. *Cellular Physiology and Biochemistry*.

[27] Samame Perez-Vargas J. C., Biondani P., Maggi C. (2013). Role of cMET in the development and progression of colorectal cancer. *International Journal of Molecular Sciences*.

[28] Liebermann D. A., Hoffman B. (2007). Gadd45 in the response of hematopoietic cells to genotoxic stress. *Blood Cells, Molecules and Diseases*.

[29] Bouchier-Hayes L., Martin S. J. (2002). CARD games in apoptosis and immunity. *EMBO Reports*.

[30] Shi F., Kong B. W., Song J. J., Lee J. Y., Dienglewicz R. L., Erf G. F. (2012). Understanding mechanisms of vitiligo development in Smyth line of chickens by transcriptomic microarray analysis of evolving autoimmune lesions. *BMC Immunology*.

[31] Jin Y., Birlea S. A., Fain P. R., Spritz R. A. (2007). Genetic variations in *NALP1* are associated with generalized vitiligo in a Romanian population. *The Journal of Investigative Dermatology*.

[32] Beg A. A., Baltimore D. (1996). An essential role for NF-*κ*B in preventing TNF-*α*-induced cell death. *Science*.

[33] Fan Y. L., Chen L., Wang J., Yao Q., Wan J. Q. (2013). Over expression of PPP2R2C inhibits human glioma cells growth through the suppression of mTOR pathway. *FEBS Letters*.

[34] Yamaguchi Y., Morita A., Maeda A., Hearing V. J. (2009). Regulation of skin pigmentation and thickness by Dickkopf 1 (DKK1). *The Journal of Investigative Dermatology Symposium Proceedings*.

[35] Xie J., Xiang D. B., Wang H. (2012). Inhibition of Tcf-4 induces apoptosis and enhances chemosensitivity of colon cancer cells. *PLoS One*.

[36] Singh R. K., Lee K. M., Vujkovic-Cvijin I. (2016). The role of IL-17 in vitiligo: a review. *Autoimmunity Reviews*.

[37] Kotobuki Y., Tanemura A., Yang L. (2012). Dysregulation of melanocyte function by Th17-related cytokines: significance of Th17 cell infiltration in autoimmune vitiligo vulgaris. *Pigment Cell & Melanoma Research*.

[38] Singh S., Singh U., Pandey S. S. (2012). Serum concentration of IL-6, IL-2, TNF-*α*, and IFN*γ* in vitiligo patients. *Indian Journal of Dermatology*.

[39] Yu H. S., Chang K. L., Yu C. L. (1997). Alterations in IL-6, IL-8, GM-CSF. TNF-*α*, and IFN-*γ* release by peripheral mononuclear cells in patients with active vitiligo. *The Journal of Investigative Dermatology*.

[40] Singh M., Mansuri M. S., Parasrampuria M. A., Begum R. (2016). Interleukin 1-*α*: a modulator of melanocyte homeostasis in vitiligo. *Biochemistry & Analytical Biochemistry*.

[41] Patel K. R., Singam V., Rastogi S., Lee H. H., Silverberg N. B., Silverberg J. I. (2019). Association of vitiligo with hospitalization for mental health disorders in US adults. *Journal of the European Academy of Dermatology and Venereology*.

[42] Plonka P. M., Passeron T., Brenner M. (2009). What are melanocytes really doing all day long…?. *Experimental Dermatology*.

[43] Sarkar S., Sarkar T., Sarkar A., Das S. (2018). Vitiligo and psychiatric morbidity: a profile from a vitiligo clinic of a rural-based tertiary care center of eastern India. *Indian Journal of Dermatology*.

[44] Blanchet P., Bebin M., Bruet S. (2017). MYT1L mutations cause intellectual disability and variable obesity by dysregulating gene expression and development of the neuroendocrine hypothalamus. *PLoS Genetics*.

[45] Liang X., Song M. R., Xu Z. (2011). Isl1 is required for multiple aspects of motor neuron development. *Molecular and Cellular Neurosciences*.

[46] Zhang H., Wang W. P., Guo T. (2009). The LIM-homeodomain protein ISL1 activates insulin gene promoter directly through synergy with BETA2. *Journal of Molecular Biology*.

[47] Shimomura H., Sanke T., Hanabusa T., Tsunoda K., Furuta H., Nanjo K. (2000). Nonsense mutation of islet-1 gene (Q310X) found in a type 2 diabetic patient with a strong family history. *Diabetes*.

[48] Munoz-Munoz J. L., Acosta-Motos J. R., Garcia-Molina F. (2010). Tyrosinase inactivation in its action on dopa. *Biochimica et Biophysica Acta (BBA) - Proteins and Proteomics*.

[49] Bolger A. M., Lohse M., Usadel B. (2014). Trimmomatic: a flexible trimmer for Illumina sequence data. *Bioinformatics*.

[50] Kopylova E., Noe L., Touzet H. (2012). SortMeRNA: fast and accurate filtering of ribosomal RNAs in metatranscriptomic data. *Bioinformatics*.

[51] Zhbannikov I. Y., Hunter S. S., Foster J. A., Settles M. L. SeqyClean: a pipeline for high-throughput sequence data preprocessing.

[52] Dobin A., Davis C. A., Schlesinger F. (2013). STAR: ultrafast universal RNA-seq aligner. *Bioinformatics*.

[53] Liao Y., Smyth G. K., Shi W. (2014). featureCounts: an efficient general purpose program for assigning sequence reads to genomic features. *Bioinformatics*.

[54] Ritchie M. E., Phipson B., Wu D. (2015). *limma* powers differential expression analyses for RNA-sequencing and microarray studies. *Nucleic Acids Research*.

[55] Huang D. W., Sherman B. T., Tan Q. (2007). The DAVID gene functional classification tool: a novel biological module-centric algorithm to functionally analyze large gene lists. *Genome Biology*.

