# Supplementary material for: RNA-seq Reveals Dysregulation of Novel Melanocyte Genes upon Oxidative Stress: Implications in Vitiligo Pathogenesis
Source: Oxid Med Cell Longev. 2019 Dec 4;2019:2841814. doi: 10.1155/2019/2841814 (PMC6913168; doi:10.1155/2019/2841814)
Supplement: Supplementary Materials — Supplementary Figure-1: comparison of the DEGs detected by three methods: Venn diagram of DEGs detected by the edgeR, DESeq, and limma. Supplementary Figure-2: the hierarchical clustering of top 100 differentially expressed genes (A): control vs. 100 μM H2O2 (B): control vs. 350 μM H2O2 at 48 h treatment and control. Red color is the upregulated and green color is the downregulated genes. Supplementary Figure-3: GO enrichment analysis of DEGs (A): 100 μM H2O2 and control samples at 48 h; (B): 350 μM H2O2 and control samples at 48 h, according to the DAVID bioinformatics tool. Supplementary Figure-4: biological pathway enrichment analysis of DEGs (A): 100 μM H2O2 and control for 48 h; (B): 350 μM H2O2 and control for 48 h using the DAVID bioinformatics tool. Supplementary Figure-5: PIG1 cells were treated with 250 μM H2O2 for 48 h, and cell viability was measured by trypan blue dye exclusion assay (A). Relative gene expression of indicated genes was measured using quantitative RT-PCR (B). GAPDH and beta-actin were used as internal controls separately. Supplementary Table-1: list of differentially regulated selected apoptotic proteins after 24 h of treatment with H2O2. [file 2841814.f1.pdf]

Supplementary Figure-1

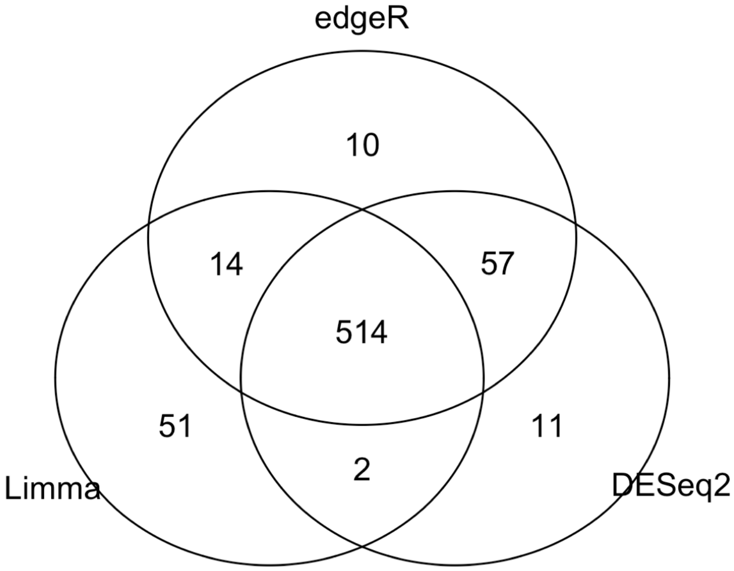

Supplementary Figure-2

A

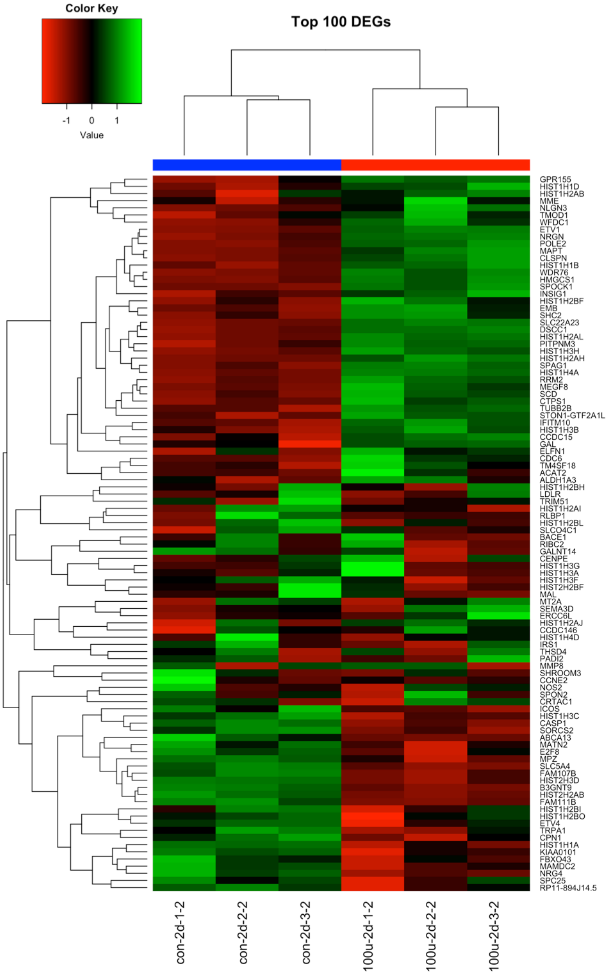

B

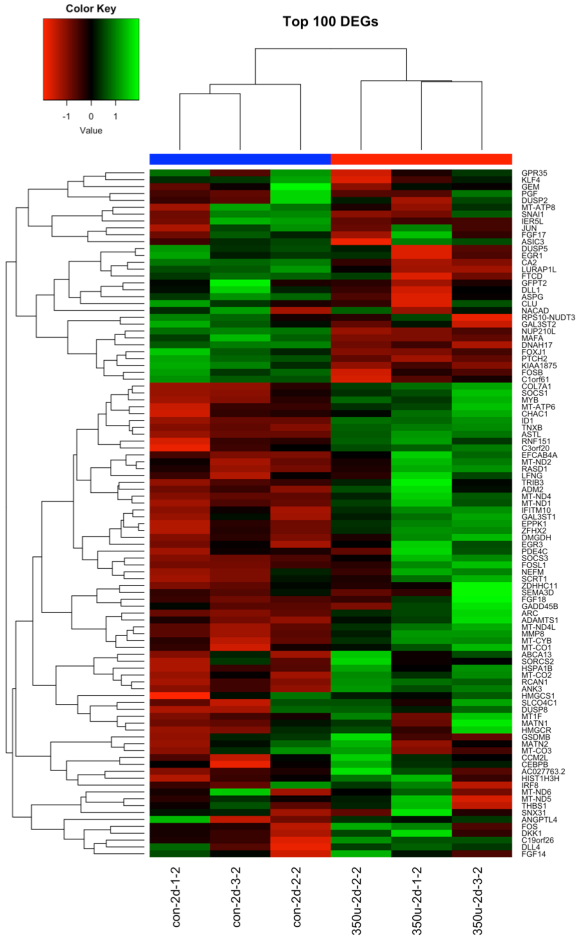

Supplementary Figure-3

Con vs 100 µM- 48 h

Molecular Function

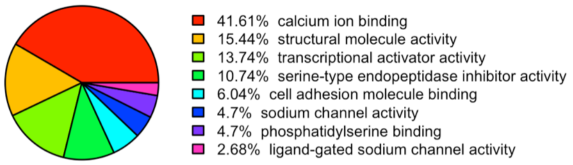

Biological Process

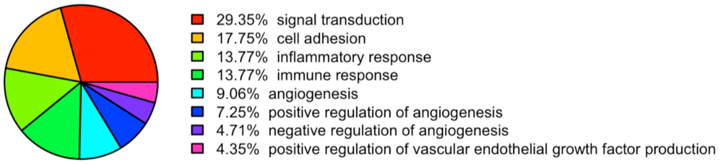

Cellular Component

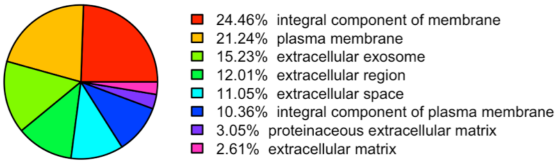

Con vs 350 µM-48 h

Molecular Function

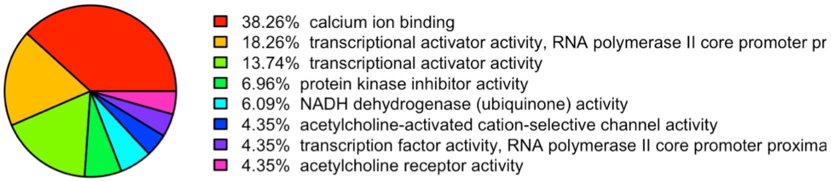

Biological Process

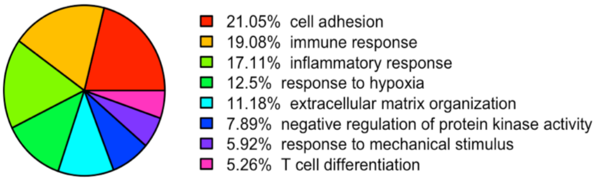

Cellular Component

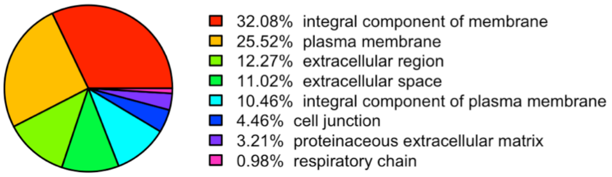

Supplementary Figure-4

Con vs 100 µM- 48 h

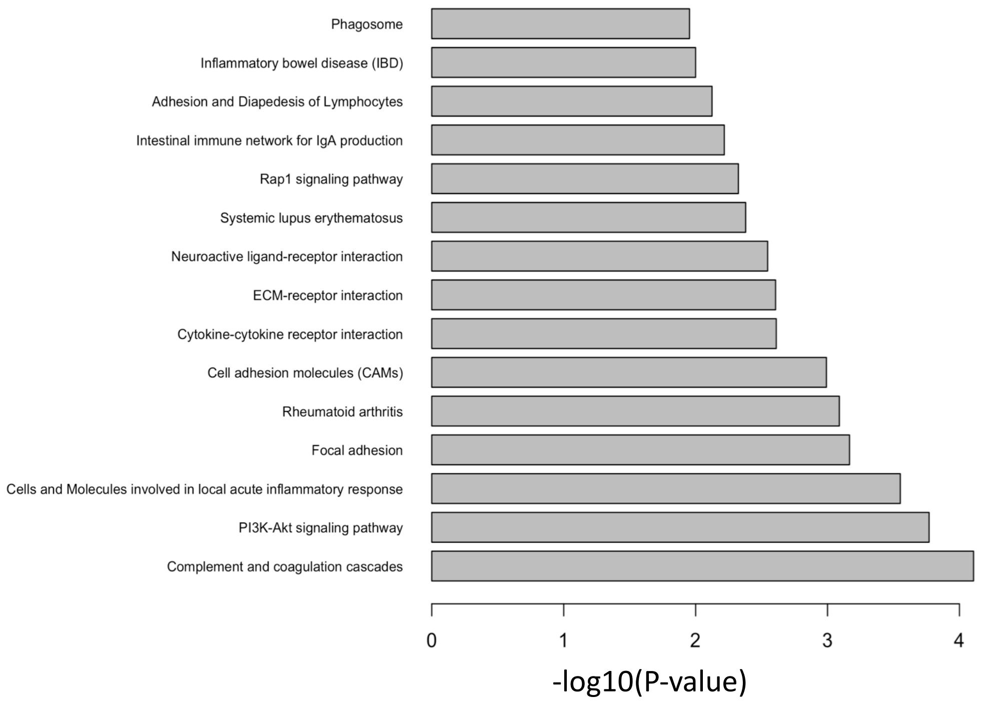

Con vs 350 µM-48 h

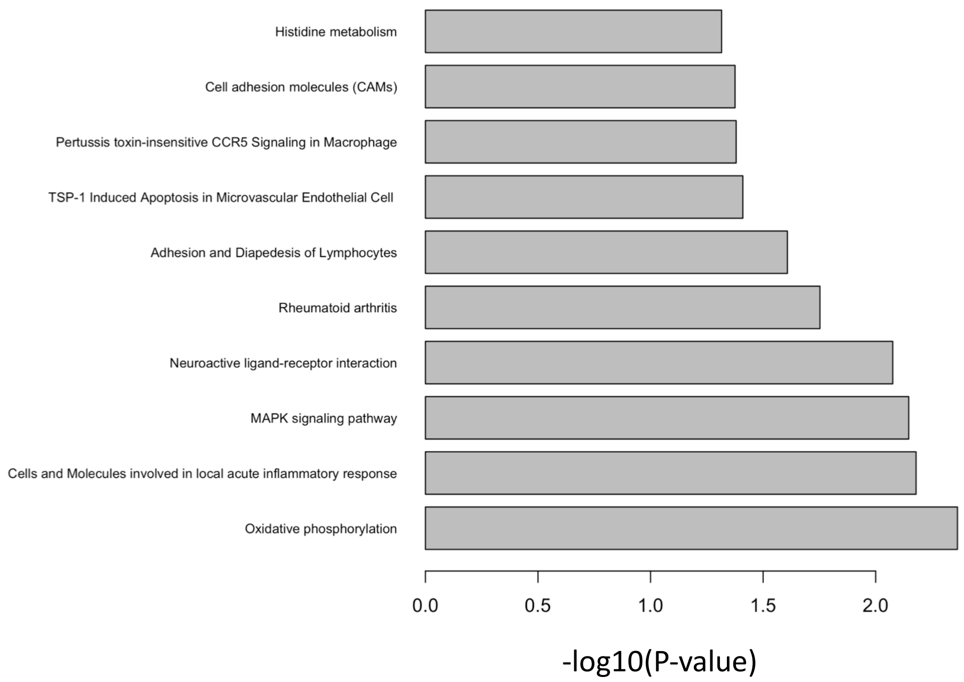

Supplementary Figure-5

A

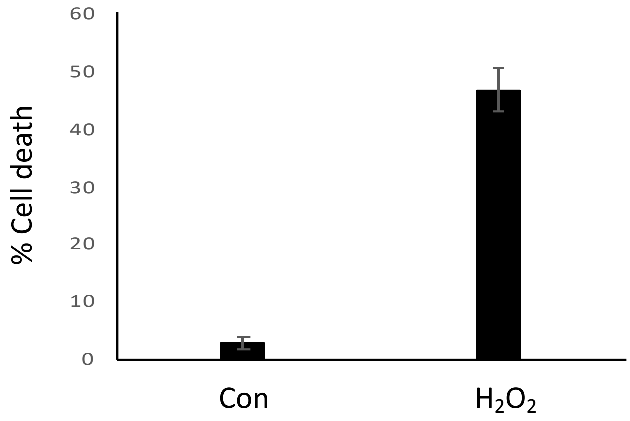

B

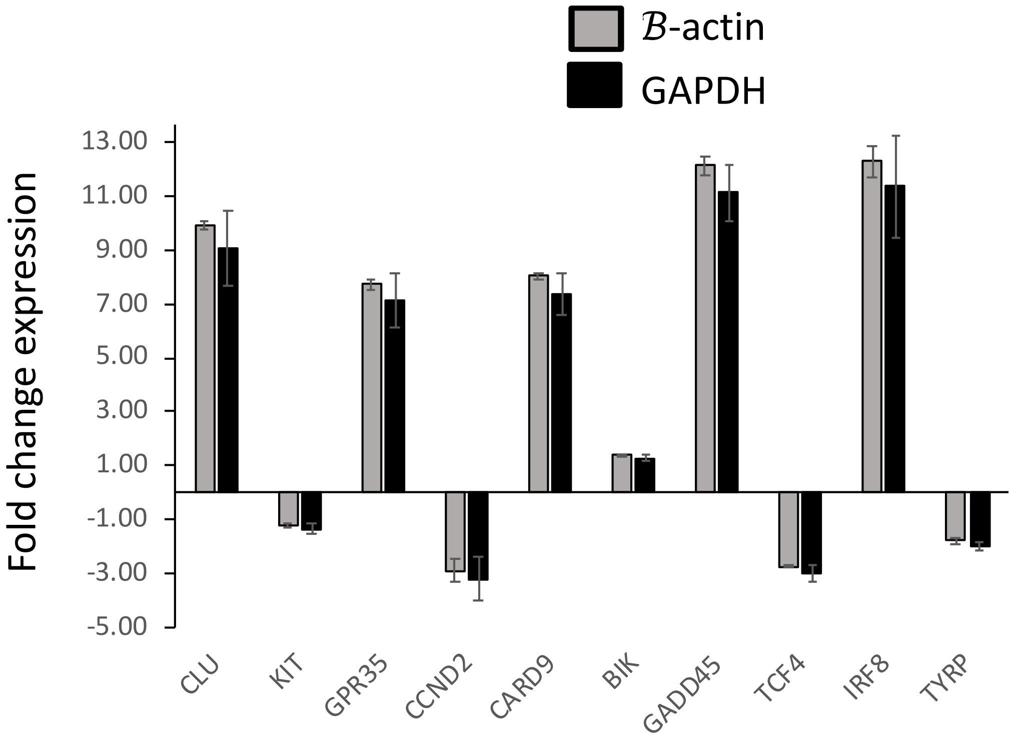

SUPPLEMENTARY INFORMATION:

Supplementary Figure-1: Comparison of the DEGs detected by three methods: Venn diagram of DEGs detected by the edgeR, DESeq and Limma.

Supplementary Figure-2: The hierarchical clustering of top 100 differentially expressed genes **(A):** Control vs 100 µM H_2O_2 **(B):** Control vs 350 µM H_2O_2 at 48 h treatment and control. Red color is the upregulated and Green color is the downregulated genes.

Supplementary Figure-3: GO enrichment analysis of DEGs **(A):** 100 µM H_2O_2 and control samples at 48 h; **(B):** 350 µM H_2O_2 and control samples at 48 h, according to DAVID Bioinformatics tool.

Supplementary Figure-4: Biological pathway enrichment analysis of DEGs **(A):** 100 µM H_2O_2 and control for 48 h; **(B):** 350 µM H_2O_2 and control for 48 h using DAVID bioinformatics tool.

Supplementary Figure-5: PIG1 cells were treated with 250 µM H_2O_2 for 48 h and cell viability was measured by trypan blue dye exclusion assay (A). Relative gene expression of indicated genes was measured using quantitative RT-PCR (B). GAPDH and beta-actin were used as internal controls separately.

Supplementary Table-1: List of differentially regulated selected apoptotic proteins after 24 h of treatment with H_2O_2

**Putative Function**	**Genes**	**Log2 Fold Change**
Apoptosis	TNFRSF9	+3.4
	TNFRSF8	+3.8
	TNFRSF1B	+1.5
	TNFRSF10A	+1.2
	TNFRSF11B	+2.5
	TNFRSF12A	+3.2
	TNFRSF13C	+1.2
	CARD9	+2.6
	CARD11	+3.1
	NALP3/NLRP3	+5.0
